# Polymorphisms of IL12RB2 May Affect the Natural History of Primary Biliary Cholangitis: A Single Centre Study

**DOI:** 10.1155/2017/2185083

**Published:** 2017-02-19

**Authors:** Urszula Wasik, Ewa Wunsch, Gary L. Norman, Eirini I. Rigopoulou, Dimitrios P. Bogdanos, Piotr Milkiewicz, Małgorzata Milkiewicz

**Affiliations:** ^1^Department of Medical Biology, Pomeranian Medical University, Szczecin, Poland; ^2^Translational Medicine Group, Pomeranian Medical University, Szczecin, Poland; ^3^Inova Diagnostics, San Diego, CA, USA; ^4^The Faculty of Medicine, School of Health Sciences, University of Thessaly, Larissa, Greece; ^5^Liver Sciences, Division of Liver Transplantation and Mucosal Biology, King's College London School of Medicine, Denmark Hill Campus, London SE5 9RS, UK; ^6^Liver and Internal Medicine Unit, Department of General, Transplant and Liver Surgery, Medical University of Warsaw, Warsaw, Poland

## Abstract

*Background*. Recent GWAS in primary biliary cholangitis (PBC) showed strong associations with SNPs located within interleukin-12 receptor (IL12R) beta-2* (IL12RB2)* gene.* Aims*. We assessed whether genetic variation of* IL12RB2* is associated with laboratory and clinical features of PBC.* Methods*. Genomic DNA was isolated from 306 patients with PBC and 258 age/gender-matched controls. PBC-specific anti-mitochondrial antibodies (AMA) were tested in all subjects by ELISA. Two SNPs, rs3790567 and rs6679356, of* IL12RB2* were genotyped using the MGB-TaqMan SNP assay.* Results*. Despite comparable age at diagnosis of cirrhotic and noncirrhotic PBC patients, allele A of rs3790567 and allele C of rs6679356 were overrepresented in the former rather than the latter group (*p* = 0.0009 and *p* = 0.002, resp.). The risk of cirrhosis at presentation increased when allele A and allele C coexisted. AMA-M2 titres were significantly higher in AA homozygotes of rs3790567 compared to GG homozygotes (132 ± 54 versus 103 ± 62, *p* = 0.02) and in rs6679356 when C allele was present (*p* = 0.038). There were no other significant associations between* IL12RB2 *polymorphisms and laboratory or clinical features.* Conclusion*. In this first study analyzing phenotypic features of PBC carriers of the* IL12RB2 *polymorphisms, we found that carriers are more frequently cirrhotic at diagnosis and have significantly higher titres of AMA.

## 1. Introduction

Interleukin-12 (IL-12) belongs to the IL-12 family cytokines along with IL-23, IL-27, and IL-35. IL-12 is a heterodimer comprised of two subunits: a 35 kDa (p35) light chain encoded by* IL12A* gene and a 40 kDa heavy chain (p40), encoded by* IL12B* gene. The synthesis and secretion of IL-12 occur* via* stimulation of Toll-like receptors expressed by antigen presenting cells (APCs) in response to microbial constituents [1, 2]. IL-12 exerts its immunobiological action through its binding to specific IL-12 receptors (IL-12Rs), termed IL-12R*β*1 and IL-12R*β*2, which are mapped on chromosomes 19p13.1 and 1p31.2, respectively [1–3]. Secreted IL-12 targets naive T cells, NK cells, and NKT cells and promotes their proliferation and differentiation. The most significant function of IL-12 is its involvement in the differentiation of T-helper 1 from naive CD4+ T cells resulting in the secretion of the proinflammatory interferon-*γ* (IFN-*γ*) cytokine [1–4].

Primary biliary cholangitis (PBC), formerly known as primary biliary cirrhosis [5, 6], is an autoimmune cholestatic liver disease, affecting up to 1 in 1000 women over 40 years of age with complaints such as fatigue and/or pruritus [7–9]. The hallmark of PBC is the presence of high-titre anti-mitochondrial autoantibodies (AMA) and the accumulation of antigen-specific autoreactive B and (CD4+ and CD8+) T cells targeting biliary epithelial cells [10]. Studies on patients with PBC, experimental work on animal models, and in vitro studies indicate a potential role of the IL-12 pathway in the development of autoimmune cholangitis [11, 12]. There is evidence for a pathogenic role for IL-12B in experimental murine model of PBC. It has been demonstrated that deletion of the IL-12B on the transforming growth factor *β* receptor II dominant negative (dnTGF*β*RII) murine model of PBC results in the amelioration of cholangitis [13].

Genetic studies provided additional support in favour of the instrumental role of IL-12 in the immunopathogenesis of PBC. Genome wide association studies (GWAS) provided evidence that PBC displays a strong association with SNPs located within the* IL12A* as well as within* IL12RB2* gene [14, 15]. The involvement of the IL-12 pathway in PBC could be indirectly supported by associations found between PBC and several SNPs across the* STAT4 *gene, which encodes an effector that is essential for interleukin-12 signaling [16]. To better understand the extent of involvement of IL-12 in PBC, we analyzed the potential association between two SNPs located in the IL-12R*β*2 gene and phenotypic features of PBC in a well-defined cohort of patients with PBC.

## 2. Methods

### 2.1. Patients and Controls

All patients with PBC fulfilled the diagnostic criteria established by the European Association for the Study of Liver (EASL) [17]. The study was performed in a homogenous group of Caucasian patients with PBC who were treated in two Polish liver transplant centres, in Szczecin and in Warsaw. Patients recruited at both sites did not differ in terms of demographic, clinical, and biochemical characteristics (data not shown). One hundred and seven (36.5%) subjects were diagnosed with liver cirrhosis, by either liver biopsy or imaging screening. Data on cirrhosis status were not available in 28 patients. Clinical and laboratory features of analyzed subjects are summarized in Table 1. Health-related quality of life (HRQoL) was assessed with disease-specific questionnaires (PBC-40 and PBC-27) [18]. PBC-specific AMA were tested by ELISA (Inova Diagnostics, San Diego, CA, USA).

Control group was comprised of 258 age- and gender-matched subjects without liver diseases. These controls were also tested for AMA using the same ELISA.

### 2.2. Genotyping

Genomic DNA from peripheral blood mononuclear cells was isolated using the DNeasy Blood & Tissue Kit (Qiagen). Oligonucleotide primers and TaqMan probes for IL-12R*β*2 polymorphisms (rs3790567, rs6679356) were designed and synthesized by Applied Biosystems. The fluorescence data were analyzed with allelic discrimination 7500 Software v.2.0.2.

### 2.3. Ethics

Appropriate informed consent was obtained from each patient included in the study. The study protocol was approved by the Ethics Committee of Pomeranian Medical University and Medical School of Warsaw. They conformed to the Ethical Guidelines of the 1975 Declaration of Helsinki (6th revision, 2008).

### 2.4. Statistical Analysis

Data are shown as means and standard deviations. All statistical analyses (chi-square, odds ratios, and confidence intervals) were carried out using StatView software (Cary, NC, USA). The genotype and allelic frequencies were compared between patients and controls using Fisher's PLSD test. The analysis of genotype frequency within PBC patients in regard to the clinical characteristics was performed using Fisher's PLSD test. The general genotype-dependent survivals were estimated using the Kaplan-Meier survival curves.

## 3. Results

### 3.1. Distribution of* IL12RB2* Polymorphisms in Patients with PBC and Controls

The allele A of rs3790567 and allele C of rs6679356 were overrepresented in PBC patients compared to controls, 30.4% versus 20.7% (*p* = 0.0009) and 25% versus 17.2% (*p* = 0.0022), respectively, confirming a significant impact of* IL12RB2* polymorphisms on PBC susceptibility (Table 2).

### 3.2. Association of rs3790567 and rs6679356 with Liver Cirrhosis

The frequency distributions of the rs3790567 and rs6679356 genotype in cirrhotic and noncirrhotic patients are shown in Table 3. Allelic analysis demonstrated that allele A of rs3790567 and allele C of rs6679356 were overrepresented in patients with histological and/or clinical features of liver cirrhosis (38% versus 25%, *p* = 0.001 and 31% versus 20.8%, *p* = 0.013, resp.). Moreover, subjects who were GG homozygote of the rs3790567 were overrepresented in noncirrhotic group (*p* = 0.003), similarly to subjects who were TT homozygote of rs6679356 (*p* = 0.009). The risk of cirrhosis increased when A (rs3790567) allele and C (rs6679356) allele coexisted (*p* = 0.007).

### 3.3. The Association of rs3790567 and rs6679356 with Features of PBC

A positive association between* IL12RB2* polymorphisms and the PBC-specific AMA-M2 concentration was found in patients with PBC. The AA-carriers of rs3790567 were characterized by an increased concentration of AMA-M2 in comparison to GG-carriers (*p* = 0.0231) (Table 4). The association of rs6679356 genotypes with higher AMA-M2 concentrations has not been observed. However, higher serum AMA-M2 was associated with the presence of allele C (*p* = 0.0384). No other associations between polymorphism distribution and clinical or laboratory features of PBC were found.

## 4. Discussion

IL-12 activates through its receptor's subunits a cascade of signaling factors such as NFK*β* and STAT4 to promote the production of proinflammatory Th1-type cytokines including TNF*α* and IFN*γ*, as well as to enhance the cytotoxic response of NK, NKT, and CD8+ T cells [19]. Genetic association data that have emerged from GWAS highlighted a relationship between the IL-12/STAT4/Th1 pathway and PBC [14, 15, 20, 21]. Two SNPs, namely, rs3790567 and rs6679356, in the intronic regions downstream of* IL12RB2* showed strong associations with PBC in a Canadian PBC cohort when compared to controls [20]. Another GWAS on Italian PBCs confirmed disease's association with rs3790567 [14].

Such GWAS are by nature unable to assess whether SNPs are associated with features of the disease, namely, presence of cirrhosis at diagnosis, fibrosis stage, liver biochemical indexes, and response to treatment or magnitude of autoantibody response. In the present study, we evaluated the association between* IL12RB2* polymorphisms and PBC in a cohort of Caucasian individuals with PBC and unaffected controls. As clinicians are more interested to know whether specific genetic markers are associated with features of the disease, we analyzed the potential association between rs3790567 and rs6679356 and clinical or laboratory features of the disease in our PBC cohort.

Our study, including a homogenous cohort of more than 300 Caucasian patients with PBC, confirmed the GWAS findings; the allele A of rs3790567 and allele C of rs6679356 are indeed overrepresented in PBC compared to disease-free controls. In view of the aforementioned associations, we considered our cohort an adequate population to further investigate the potential relationship between such polymorphisms and laboratory as well as clinical features of the disease.

In PBC, small and medium-size intrahepatic bile ducts are destroyed by an autoimmune attack which if left untreated could lead to liver cirrhosis in a proportion of patients with PBC. At times, patients present with cirrhosis and a diagnosis of PBC is made at presentation, based on laboratory, immunological, and clinical features compatible with the autoimmune cholestatic nature of the disease. Our allelic analysis demonstrated that the allele A of rs3790567 and the allele C of rs6679356 significantly increased risk of cirrhosis at the diagnosis. The IL-12 signaling pathway is a key player in the effector mechanisms that lead to biliary destruction and subsequent liver cirrhosis. IL-12R*β*2 is upregulated by IFN*γ* triggered by IL-12; thus in this case cytokine-receptor interaction provides a positive feedback loop further promoting biliary epithelial cell damage. Since IL-12-dependent, self-perpetuating autoimmune mechanism appears to be responsible for cholangiocyte destruction, it is reasonable to expect a more progressive pace of the disease in patients who carry specific* IL12RB2* polymorphisms.

The characteristic immunological hallmark of PBC is the presence of disease-specific M2-AMA in serum of affected patients [22]. More than 90% of PBC patients have detectable AMA, but their titres vary amongst affected individuals. Our data show that analyzed* IL12RB2 *polymorphisms were associated with significantly higher AMA-M2 titres. IL-12 is secreted by activated APCs. It is likely that their activation occurs* via* release of E2 subunit mitochondrial pyruvate dehydrogenase autoantigen (PDC-E2) during apoptosis of biliary epithelial cells (BECs) [23, 24]. PDC-E2 is endocytosed by APC and this endocytosis leads to APC maturation and IL-12 secretion which provides the cytokine milieu which is essential for the maintenance of the continuum of proinflammatory T-helper type 1 (Th1) induction arising from naive CD4+ T through antigen presentation; such antigen-specific CD4 T cells help plasmocytes to produce high-titre AMA targeting PDC-E2. IL-12R*β*2 appears to be pivotal for the continuation of this deleterious positive feedback. Though our study is the first to identify* IL12RB2 *polymorphisms positively associated with the magnitude of AMA response, this is not the first time that an association between* IL12RB2 *and high-titre autoantibodies has been found in autoimmune diseases, providing a link between the IL-12/IL-12R axis and the scale of the autoimmune attack at the humoral level. This is not the first time that an association between autoantibody status and IL12-IL12R/STAT4 polymorphisms has been reported. In fact, Joshita et al. [16] have found an association between three STAT4 PBC risk alleles (rs7574865, rs8179673, and rs10181656) with ANA status (but not with AMA positivity) in Japanese patients with PBC; IL12R polymorphisms do not appear to be risk alleles in non-Caucasian patients, and in particular those originated from Japan, and this may explain why associations were not reported by the former study between AMA or ANA with IL12R alleles. In systemic lupus erythematosus, the presence of anti-dsDNA antibodies is also associated with rs7574865 within* STAT4*, further highlighting the close relationship between the immunogenetic make-up of the autoimmune disease and pathognomonic immunological features, such as disease-related autoantibodies [25].

We found no association between analyzed polymorphisms and liver biochemistry indexes; this is not unexpected as liver cirrhosis is a “burnt out” condition characterized by low aminotransferase levels. We have recently published that patients with PBC have impaired HRQoL, irrespectively of whether they are cirrhotic or not [18]; thus an association between the analyzed polymorphisms and HRQoL measures depicted by the PBC-40 or PBC-27 questionnaires would be rather unlikely; this appears to be the case, as our analysis failed to identify significant associations.

In conclusion, we have confirmed the relationship of* IL12RB2* polymorphisms with PBC susceptibility. Moreover, we have provided evidence that* IL12RB2* polymorphisms are associated with liver cirrhosis and an increased concentration of disease-specific AMA in sera of PBC patients. Larger, multicentre studies are warranted to assess the diagnostic and prognostic significance of our findings in a more comprehensive manner.

## Figures and Tables

**Table 1:** Clinical and laboratory data in patients with primary biliary cholangitis (PBC).

	PBC	PBC		*p* value (cirrhotic versus noncirrhotic)
		Noncirrhotic	Cirrhotic	
Age (years)	53 ± 11	51 ± 9	53 ± 10	NS
Gender (female/male)	277/29	152/6	89/18	*p* = 0.004
Liver cirrhosis (yes/no/unknown)	107/158/41	158	107	—
Death or OLTx (yes/no/unknown)	66/214/26	17/133	42/61	*p* < 0.001
UDCA response (yes/no/unknown)	81/120/105	46/47	24/56	*p* = 0.01
ALP (IU/l; mean ± SD; normal: 30–120)	363 ± 313	149 ± 306	102 ± 339	NS
ALT (IU/l; mean ± SD; normal: 3–30)	104 ± 122	110 ± 126	97 ± 123	NS
AST (IU/l; mean ± SD; normal: 3–30)	95 ± 112	85 ± 104	105 ± 123	NS
*γ*-GT (IU/l; mean ± SD; normal: 3–30)	365 ± 417	360 ± 437	342 ± 380	NS
Bilirubin (mg/dl; mean ± SD; normal: 0.2–1.0)	3.1 ± 5.6	1.6 ± 2.9	5.2 ± 7.9	NS

**Table 2:** Distribution of *IL12RB2* polymorphisms (rs3790567, rs6679356) in PBC patients and control subjects.

SNP	Genotype	Count of alleles/genotypes		Fisher exact *p* value	*χ* ^2^	OR
		Control (*n* = 258) (%)	PBC (*n* = 306) (%)			
rs3790567	A/G	107/409 (20.7/79.3)	186/426 (30.4/69.6)	0.0009	11.5	1.7 [1.3–2.2]
	AA	11 (4.2)	29 (9.5)	0.02	5.8	2.3 [1.1–4.8]
	AG	85 (33)	128 (42)	0.02	4.7	1,5 [1.0–2.1]
	GG	162 (62.8)	149 (48.5)	0.0009	11.3	0.6 [0.4–0,8]

rs6679356	C/T	89/427 (17.2/82.8)	153/459 (25/75)	0.002	9.5	1.6 [1.2–2.1]
	CC	11 (4.2)	22 (7.2)	NS	2.2	1,7 [0.8–3.6]
	CT	67 (26)	109 (35.6)	0.01	6.1	1,6 [1.1–2.3]
	TT	180 (69.8)	175 (57.2)	0.03	5.1	0,6 [0.4–0.8]

**Table 3:** The *IL12RB2* polymorphisms (rs3790567, rs6679356) association with cirrhosis in PBC.

SNP	Genotype	Cirrhosis (*n* = 276)		Fisher exact *p* value	*χ* ^2^	OR
		No (%)	Yes (%)			
rs3790567	A/G	83 (25%)/247 (75%)	84 (38%)/138 (62%)	0.001	10.12	1.8 [1.3–2.6]
	AA	12 (7.3%)	16 (14.4%)	NS	3.71	2.1 [0.9–4.7]
	AG	59 (35.7%)	52 (46.9%)	NS	3.39	1.6 [0.9–2.6]
	GG	94 (57%)	43 (38.7%)	0.003	8.82	0.5 [0.3–0.8]

rs6679356	C/T	69 (20.8%)/263 (79.2%)	68 (31%)/152 (69%)	0.013	7.27	1.7 [1.1–2.5]
	CC	10 (6%)	12 (10.9%)	NS	2.15	1.9 [0.8–4.6]
	CT	49 (29.5%)	44 (40%)	NS	3.25	1.6 [0.9–2.6]
	TT	107 (64.5%)	54 (49.1%)	0.009	6.43	0.5 [0.3–0.9]

**Table 4:** The *IL12RB2* polymorphisms (rs3790567, rs6679356) association with M2-AMA concentration in patients with PBC.

SNP	Genotype	Count of genotypes (*n* = 295)	AMA level-mean [IU/l]	*p*
rs3790567	A/G	177/413		NS
	AA	27	132 ± 54	AA versus GG *p* = 0.023
	AG	123	107 ± 62	
	GG	145	103 ± 62	

rs6679356	C/T	147/443		0.0384
	CC	21	128 ± 60	CC versus TT NS
	CT	105	113 ± 60	
	TT	169	101 ± 62	

AMA: anti-mitochondrial antibody.
